# Long-term Outcome after Robotic-assisted Gastroplication in Adolescents: Hunger Hormone and Food Preference Changes Two Case Reports

**DOI:** 10.4274/jcrpe.2283

**Published:** 2016-06-06

**Authors:** Valeria Calcaterra, Hellas Cena, Maria Luisa Fonte, Mara De Amici, Matteo Vandoni, Michela Albanesi, Gloria Pelizzo

**Affiliations:** 1 Fondazione IRCCS Policlinico San Matteo, University of Pavia and Department of Maternal and Children’s Health, Department of Internal Medicine, Pediatric Unit, Pavia, Italy; 2 University of Pavia, Department of Public Health, Neurosciences, Experimental and Forensic Medicine, Section of Human Nutrition, Pavia, Italy; 3 Fondazione IRCCS Policlinico San Matteo, Immuno-Allergy Laboratory, Clinic of Pediatric, Pavia, Italy; 4 University of Pavia, Department of Public Health and Neuroscience, Pavia, Italy; 5 Fondazione IRCCS Policlinico San Matteo and University of Pavia, Department of Maternal and Children’s Health, Pediatric Surgery Unit, Pavia, Italy; 6 Equal contribution

**Keywords:** Robotic surgery, gastroplication, ghrelin, leptin, adolescent, food choices, eating behavior

## Abstract

Weight loss surgery (WLS) is efficacious for long-term weight reduction and decreases overall mortality in severely obese patients. The mechanisms implicated in long-term weight loss are not fully understood. Proposed mechanisms include changes in gut hormones and brain regulation of appetite and satiety. We aimed to investigate the long-term ghrelin and leptin profiles and changes in food preference and eating behavior after WLS in adolescent patients. Two obese females aged 15 years and 14 4/12 years, who did not respond to lifestyle changes, including dietary intervention and physical exercise in combination with medical therapy, underwent robotic-assisted gastroplication. Anthropometric measurements, food habits and eating behavior, as well as metabolic and hormonal changes during long-term post-surgical follow-up were monitored. Long-term weight reduction was obtained in both patients, with a significant decrease in waist circumference. Resting energy expenditure showed a decrease over time, with a respiratory quotient that increased showing a shift from oxidation of a high-fat diet before surgery to oxidation of a mixed diet two and three years later. Both subjects improved their eating habits and lifestyle. Co-morbidity resolution was also noted. Increased pre-prandial ghrelin levels as well as higher post-prandial ghrelin and a leptin drop compared with pre-surgery values were observed in both patients. Persistent weight loss after gastroplication is associated with a favorable change in gut hormones and food preferences. The role of hormonal and sensory components in long-term results seems crucial. Particularly in adolescent patients, a multidisciplinary approach and continuous nutritional care is mandatory for weight maintenance and consolidation of changes.

## WHAT IS ALREADY KNOWN ON THIS TOPIC?

Weight loss surgery is efficacious for long-term weight reduction and decreases overall mortality in severely obese patients. The mechanisms implicated in long-term weight loss are not fully understood. Changes in gut hormones and brain regulation of appetite and satiety are proposed.

## WHAT THIS STUDY ADDS?

We reported long-term follow-up after gastroplication in two adolescents. Weight loss is associated to a favorable change in hunger hormone and food preferences. Hormonal and sensory components in the long-term results seems to be crucial.

## INTRODUCTION

Weight loss surgery (WLS) is efficacious for long-term weight reduction and decreases overall mortality in severely obese patients (1,2,3,4). The effect of WLS is probably not only due to restriction of food intake and/or malabsorption of ingested food, however, the mechanisms implicated in long-term weight loss are not fully understood. Proposed mechanisms include changes in gut hormones and brain regulation of appetite and satiety (5,6,7). Hormones such as ghrelin, leptin, peptide YY (PYY), glucagon-like peptide-1 (GLP-1), and cholecystokinin (CCK), secreted by the gastrointestinal (GI) tract, the pancreas, and by the adipose tissue, are released into the periphery in response to increased or decreased intake of nutrients and are able to act peripherally on the vagus nerve and centrally on target areas in the hypothalamus (8,9). In addition, crosstalk between the adipose tissue and the gut may also be relevant in the context of regulating energy homeostasis, satiety, and body weight. Leptin is released continuously from the adipose tissue into the circulation and acts mainly on the hypothalamus, regulating the long-term energy storage. In addition, exocrine-secreted gastric leptin is proposed to ensure proper food processing and food intake in the short term independently of adipose-derived leptin (10).

Modifications in the perception of food and hence eating behavior changes are also considered crucial in weight loss with long-term maintenance. Patients after WLS, particularly post Roux-En-Y Gastric Bypass (RYGB), report feeling less hungry, reaching satiety earlier, thus reporting a change in their taste and food choices. These changes have been strongly attributed to variations in taste processes and food reward (11,12,13). Reports on neuro-hormonal assessment and shifts in food habits after WLS of subjects in the pediatric age group are scarce (14,15).

In this paper, we report long-term ghrelin and leptin profiles and changes in food choices and eating behavior after robotic-assisted gastroplication in two adolescent patients.

## CASE REPORTS

Two adolescents, who did not respond to lifestyle changes, including dietary intervention and physical exercise in combination with medical therapy, underwent robotic-assisted gastroplication.

Patient 1, a 15-year-old obese female with a body mass index (BMI) of 38.8 kg/m2, was submitted to an eighteen-month organized and supervised lifestyle modification intervention, including family involvement and medical treatment (6 months of metformin) with no significant improvement. She had developed hyperinsulinism, hyperandrogenism, amenorrhea, ultrasound signs of Polycystic ovarian syndrome (PCOS), and hypertension with left ventricular hypertrophy.

Patient 2, a 14-year, 4-month-old obese female with a BMI of 41.2 kg/m2, had an unsuccessful outcome after 20 months of supervised lifestyle changes, including family involvement. She was noncompliant with the medical treatment prescribed (metformin). Hyperinsulinism, dyslipidemia, moderate hepatic steatosis, gastroesophageal reflux (GER), obstructive sleep apnea (OSA) were reported before surgery.

Both girls had attained skeletal and developmental maturity (final height and pubertal stage) before the surgical treatment. No major contraindications for WLS were found, including eating disorders and psychopathologies. A multidisciplinary intervention with specific nutritional, psychological, and training sessions was started two months before surgery and continued post-surgery. Both girls were prescribed moderate-low energy diets balanced in macro- and micronutrients according to our national recommendations specific for age and sex (16) and were invited to weekly nutrition education sessions intended to be informative and interactive. For both patients, the capability to commit to comprehensive medical and psychological evaluation before and after surgery as well as patient and family willingness to participate in a postoperative multidisciplinary treatment program were documented. Written informed consent was obtained prior to enrollment. Gastroplication was performed using the Robotic surgery Da Vinci system® (Intuitive Surgical, Inc., Sunnyvale, California, USA) with an 8.5 mm scope and two 5-mm operative trocars. Two rows of non-absorbable interrupted sutures (2-0 Ethibond™) were placed along the greater dissected curvature starting 1 cm below the angle of His, narrowing the stomach (80-100 mL of volume). No intra- or postoperative complications occurred. Pain control medication was only necessary for 2 days postoperatively and then stopped. The hospital stay lasted 96 hours. Proton pump inhibitors and anticoagulation were prescribed for 14 days.

During follow-up, metabolic and hormonal changes were documented. A complete nutritional assessment with anthropometric measurements, bioimpedance analysis, and indirect calorimetry (IC) was performed under standard conditions: overnight fasting, abstaining from caffeine, alcohol, nicotine, and strenuous physical activity starting the day before, as abstaining from moderate physical activity for at least 2 hours (17,18).

Food intake and eating habits were documented by the subjects keeping a daily food diary and a validated self-administered food frequency questionnaire (19,20) starting two months before surgery (T0) and repeated yearly post treatment.

Ghrelin and leptin were evaluated, before and after surgery, using a commercial enzyme immunometric assay (Human Unacylated Ghrelin, BioVendor R&D, Brno, Czech Republic) and an enzyme-linked immunosorbent assay kit (Human Leptin Immunoassay, R&D Systems, Minneapolis, MN) respectively, following the manufacturer’s instructions. The results were expressed as pg/mL.

The study was performed according to the Declaration of Helsinki. The Ethics Committee of the Fondazione IRCCS Policlinico San Matteo and Department of Internal Medicine, University of Pavia, approved the study protocol. All procedures were carried out with adequate understanding and written consent of the patients and their parents.

Nutritional assessment as well as metabolic and hormonal changes during long-term post-surgical follow-up are reported in Tables 1, 2, 3.

**Anthropometric Measurements and Food Habits and Eating Behavior**

Long-term weight reduction was obtained in both patients with a significant decrease in waist circumference (Table 1). Resting energy expenditure (REE) measured by IC also showed a significant decrease over time, with a respiratory quotient (RQ) that increased, showing a shift from oxidation of a high-fat diet before surgery to oxidation of a mixed diet two and three years later.

Coupling the measurement of body composition to that of REE expands the diagnostic potential of IC (18). Once the lean and fat compartments had been measured by bioelectrical impedance analysis, it was possible to establish on the basis of REE whether the two subjects were becoming hyper- or hypo-metabolic. The results show, as expected, a slight decrease in metabolic efficiency during the first period (1 year after surgery) and values similar to initial ones despite weight loss during the following recovery period (Table 1).

Table 2 reports the main changes after WLS for food consumption, eating behavior, and lifestyle behavior.

Both subjects improved their eating habits and changed their lifestyle behavior (Table 1).

Dietary intake was assessed using a prospective 7-day food diary. Total energy intake (kcal/day) was estimated based on the food diary and showed, though not equal, significant reductions in both subjects. Major changes were evident in particular the intake of proteins, compared to baseline (before surgery). Lipid and carbohydrate intake changed until reaching the national reference recommended dietary intake (16). Alternatively, fiber intake slowly increased by a more than expected rate, over the years (Table 3).

**Metabolic Changes and Co-Morbidities**

Patient 1 had a significant reduction in blood insulin level and a decrease in homeostatic model assessment-insulin resistance (Table 1). Resolution of hormonal and ultrasonographic features of PCOS was observed, and the girl regained normal menstrual cycles 5 months after surgery. Blood pressure also decreased to normal levels.

Patient 2 showed resolution of insulin resistance, of dysfunctional lipid metabolism and RGE (Table 1). Improvement in steatosis and OSA were also reported.

Improved social and emotional well-being and self-esteem were reported in both girls.

**Ghrelin and Leptin Profile**

**Figure 1:**
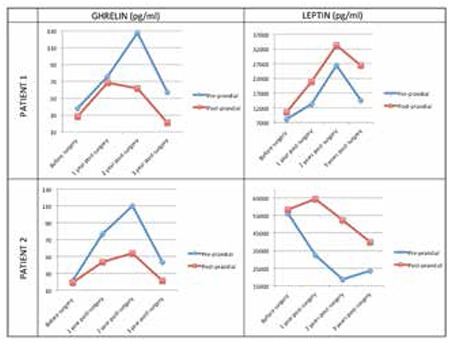
Ghrelin and leptin profiles of the two patients, before surgery and during long-term post-surgical follow-up

Increased pre-prandial ghrelin levels were observed in both patients as well as a higher post-prandial ghrelin drop and leptin increase compared with pre-operative levels (Table 1, Figure 1).

## DISCUSSION

Recent evidence highlighting the prevalence of severe obesity in the pediatric population, coupled with disappointing outcomes related to medical weight loss interventions, has led to increased interest in WLS (2,3,4). It is reported that the number of surgeries being performed in adolescents has increased 5-fold from 1997 to 2003 and tripled in 2000-2003. The number of procedures performed yearly is rising, and WLS is currently the most effective treatment for morbid obesity and improvement of comorbidities in adolescents. While pharmacological and behavioral treatments are usually associated with weight loss followed by weight regain, WLS provides weight loss for at least 15 years in obese patients (2,3,4,16).

We describe long-term weight reduction associated with resolution of comorbidities and a relevant change in neuroendocrine profile and food preferences as well as eating behaviors in two adolescents, after robotic-assisted gastroplication. This surgical approach in adolescence has the added advantages of being reversible, not requiring the use of foreign materials, and conforming to the physiological development of the individual. The clinical, metabolic, and hormonal improvements observed following a long-term follow-up confirm the effectiveness of this technique in young patients.

Gut hormones such as ghrelin, PYY, GLP-1, pancreatic polypeptide, oxyntomodulin are implicated in the short-term regulation of ingestion and adiposity signals such as insulin and leptin are involved in long-term energy homeostasis (8,10). Our data confirmed that modifications in the milieu of gut hormones is implicated in the sustained weight loss observed following WLS. After food is ingested, nutrients pass through the GI tract, stimulating the release of a range of peptide hormones. In the context of their local, central, and peripheral actions, these hormones also mediate satiety.

Ghrelin is the only known orexigenic gut hormone and it is principally secreted from X/A-like cells within gastric oxyntic glands. Ghrelin mediates its orexigenic action via stimulation of neuropeptide Y (NPY)/agouti-related peptide (AgRP), coexpressing neurons within the arcuate nucleus (ARC) of the hypothalamus. The brainstem and vagus nerve may also contribute to the effects of ghrelin on food intake.

Insulin is synthesized in the ß cells of the pancreas and is secreted rapidly after a meal, with well-characterized hypoglycemic effects. However, insulin also acts as an anorectic signal within the central nervous system (CNS); insulin receptors are widely expressed in the brain, particularly in hypothalamic nuclei, such as the ARC, dorsomedial nucleus (DMN), and paraventricular nucleus, which are involved in the control of food intake.

Leptin is predominantly secreted by adipocytes with circulating levels proportional to fat mass. It exerts its anorectic effect via the ARC, where both NPY/AgRP and pro-opiomelanocortin (POMC)/cocaine- and amphetamine-regulated transcript (CART) neurons express leptin receptors. Leptin inhibits NPY/AgRP neurons and activates POMC/CART neurons, resulting in reduced food intake and increased energy expenditure (8,10,21,22).

Obesity in children is associated with leptin and insulin resistance, manifested by reduced serum levels of ghrelin and increased leptin and insulin levels. Preprandial levels of circulating ghrelin rise, then fall rapidly in the postprandial period. Obese subjects show a less marked drop in plasma ghrelin after meal ingestion (23,24,25).

In our girls, ghrelin, leptin, and insulin levels were abnormal before surgery and a pronounced change in orexigenic and anorectic hormones was observed following WLS (4,5,6,7). The analysis showed a significant post-prandial decrease in ghrelin and an increase in leptin levels compared with pre-operative levels and confirmed the favorable impact of this surgical procedure on hunger hormonal changes. However, changes in hormones does not fully explain the magnitude of weight loss after WLS. Following WLS, body weight decreases, but body composition improves without relevant changes in the phase angle. It has been shown that phase angles are sensitive to differences in water distribution and can be used to predict % body fat mass (26). The phase angle deviates little from initial values suggesting the maintenance of metabolically active mass. Besides, the phase angle is not just an indicator of adequate nutritional status preservation but more of an overall measure of function and general health (27). Further studies are necessary to show how phase angle differs between different populations, according to age, ethnicity, and body composition (27).

Both our patients improved their eating habits by eating fruits and vegetables which they never used to eat before surgery, although their fiber intake increased more slowly than expected over the years, highlighting the obstacle of adjusting to adequate consumption of fruits and vegetables, typical of teenager habits and food dislikes.

The patients also stopped skipping breakfast in the morning, increased the number of their meals per day decreasing the food portion size and avoided spontaneous soft drinks and junk food intake. Their diet improved with changes not only in energy intake but also in macronutrient composition with an impact not only on food choice and preference but also on metabolism with a better use of energy substrates reflected in a notable increase in RQ and metabolic efficiency (18,28).

The mechanisms responsible for suppression of appetite and changes in food preferences are not well understood. Although a number of changes in food choice, taste functions, hedonic evaluation, motivation, and self-control have been documented in both humans and rodents after surgery, their importance and relative contribution to diminished appetite is still under investigation (4,5,6,7). Hedonic and sensory components like olfactory and gustatory stimuli significantly affect the appetite and taste. Recent studies have increased our knowledge on the expression of receptors being targeted by metabolic hormones and peptides governing cellular processes underlying hunger (ghrelin, NPY) and satiety (insulin, leptin) in the olfactory mucosa, the olfactory bulb, and olfactory-related brain areas (29). This chemical nutritional information alters the olfactory message and adapts the function of the olfactory system. Obesity-related changes influence the olfactory function (30). About one quarter of morbidly obese patients are hyposmic with significantly decreased discrimination and identification ability and limited gustatory function. The olfactory function can change metabolism and feeding-related behavior thus affecting the energy balance and body weight.

After laparoscopic WLS, the discrimination ability of the olfactory and gustatory functions improves (13). In our girls, no data on olfactory function were available. However, after gastroplication a shift in food preferences and development of food dislikes were observed supporting the sensory component role in long-term WLS outcomes.

It must be added that the inevitable changes due to the modifications in body weight, body shape, and body image perception create new stimuli that affect lifestyle changes and sociality.

Both of our two girl patients became more physically active and more engaging in their social relationships. Both showed a notable decrease in social isolation and reported an improvement in self-esteem and quality of life (3).

Major behavioral changes occur postoperatively. However, it is recommended to have a preoperative program to educate the subject on food choices, implement nutritional changes, and to prepare them for post-surgery modifications. The ultimate goal is to encourage the subjects to independently choose a healthier diet, which involves long-term preparation. In our patients, a very satisfactory post-operative weight loss was reported in the first and second year of follow-up, while a steady state in BMI as well as some other parameters from the second year to the third year were observed. A moderate decrease in the patients’ compliance may have contributed to this trend. The surgical procedure is only one part of the treatment plan if the goal is to obtain effective and long-lasting results leading to persistent changes.

Substantial nutrition guidance is required before and after the surgical procedure. A multidisciplinary continuous approach is required to support these patients, helping them to change their food habits, meet their protein and fluid requirements, increase their physical activity, and learn to listen to their bodies recognizing the signals for hunger and satiety. Post-surgery weight loss and food choices can be monitored by a registered dietitian or a nutritionist, while the clinician may assess the state of health and well-being and also monitor the hormonal changes. In the follow-up, micronutrients should also be evaluated and specific supplementation should be prescribed if necessary.

In conclusion, the findings in our two patients show that persistent weight loss after gastroplication is associated with a favorable change in gut hormones as well as in food preferences, eating behavior, and lifestyle. The role of hormonal and sensory components in the long-term WLS results seems crucial. We should also emphasize the need for a multidisciplinary approach and continuous nutritional education in the long-term weight maintenance and consolidation of changes, particularly in adolescent patients.

## ACKNOWLEDGMENT

The authors thank Dr. G. Nakib for surgical support, Dr. C. Torre and Dott. ssa S. Nigrisoli for technical support in the hormonal evaluation.

**Ethics**

Ethics Committee Approval: Fondazione IRCCS Policlinico San Matteo Committee (Approval number: 02-15-12), Informed Consent: It was taken.

Peer-review: External peer-reviewed.

## AUTHORSHIP CONTRIBUTIONS

Concept: Gloria Pelizzo, Valeria Calcaterra, Hellas Cena, Design: Gloria Pelizzo, Valeria Calcaterra, Hellas Cena, Data Collection or Processing: Maria Luisa Fonte, Mara De Amici, Matteo Vandoni, Michela Albanesi, Analysis or Interpretation: Gloria Pelizzo, Valeria Calcaterra, Hellas Cena, Literature Search: Valeria Calcaterra, Hellas Cena, Writing: Gloria Pelizzo, Valeria Calcaterra, Hellas Cena.

Financial Disclosure: The authors declared that this study has received no financial support.

## Figures and Tables

**Table 1:**
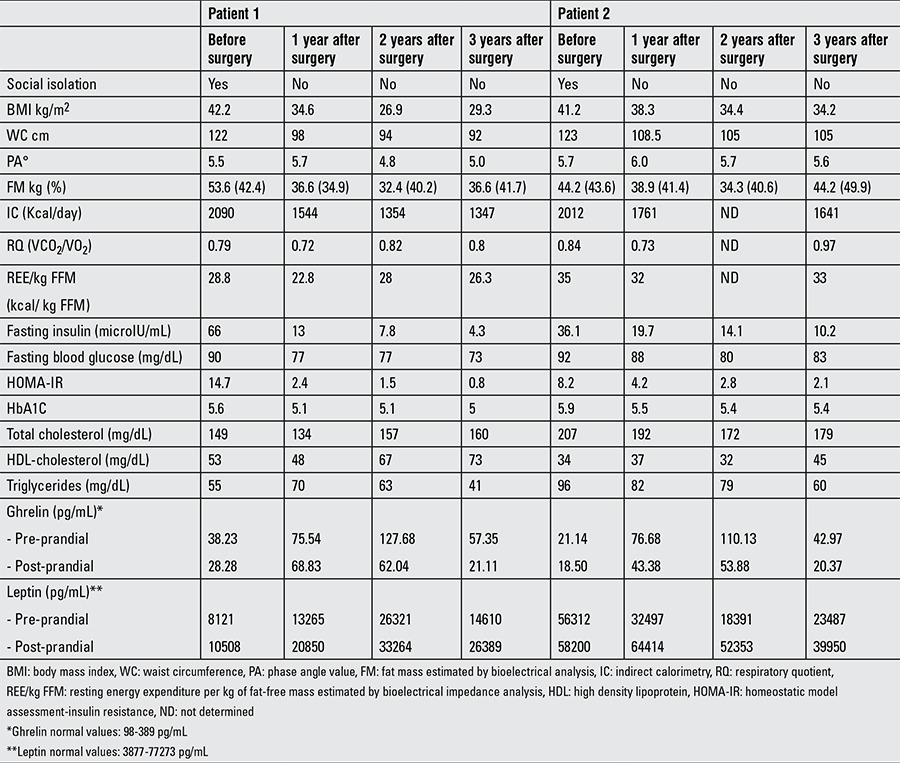
Anthropometric, metabolic, and endocrine profile of the two patients, during long-term post-surgical follow-up

**Table 2:**
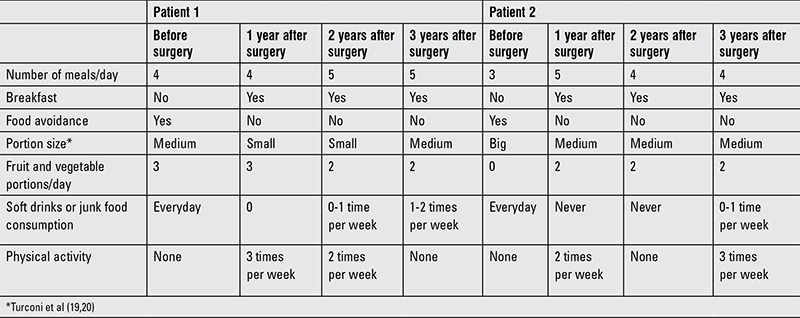
Lifestyle and food habits of the two patients, before and after surgery

**Table 3:**
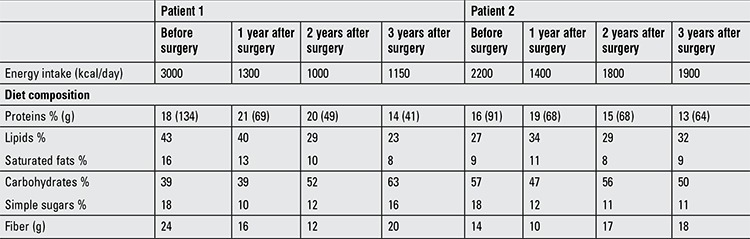
Daily energy intake and diet composition of the two patients during long-term post-surgical follow-up
